# *Spatholobus suberectus* Dunn Water Extract Ameliorates Atopic Dermatitis–Like Symptoms by Suppressing Proinflammatory Chemokine Production *In Vivo* and *In Vitro*

**DOI:** 10.3389/fphar.2022.919230

**Published:** 2022-06-20

**Authors:** Hyun-Kyung Song, Sun Haeng Park, Hye Jin Kim, Seol Jang, Taesoo Kim

**Affiliations:** ^1^ Herbal Medicine Research Division, Korea Institute of Oriental Medicine, Daejeon, South Korea; ^2^ College of Pharmacy, Seoul National University, Seoul, South Korea

**Keywords:** *Spatholobus suberectus* DUNN, atopic dermatitis, NC/Nga mice, HaCaT cells, proinflammatory chemokines

## Abstract

*S. patholobus suberectus* Dunn, a traditional Chinese herbal medicine, has various pharmacological activities, such as anti-inflammatory properties. However, to the best of our knowledge, its therapeutic effect on atopic dermatitis (AD) has not been investigated. In this study, we explored the effect of *S. suberectus* Dunn water extract (SSWex) on AD *in vivo* and *in vitro*. In *Dermatophagoides farina* extract (DfE)–treated NC/Nga mice, the oral administration of SSWex alleviated AD-like symptoms, such as ear thickness, dermatitis score, epidermal thickness, immune cell infiltration, and levels of AD-related serum parameters (immunoglobulin E, histamine, and proinflammatory chemokines). In HaCaT cells, the production of proinflammatory chemokines induced by interferon-γ (IFN-γ) and tumor necrosis factor-α (TNF-α) was inhibited by SSWex pretreatment. SSWex treatment inhibited the phosphorylation of mitogen-activated protein kinase and activation and translocation of transcriptional factors, such as signal transducer and activator of transcription 1 and nuclear factor kappa B in IFN-γ/TNF-α–stimulated HaCaT cells. These results indicate that SSWex may be developed as an efficient therapeutic agent for AD.

## Introduction

Atopic dermatitis (AD) is a chronic and allergic inflammatory skin disease caused by various environmental factors, including mite dust, smoking, and allergens. It is characterized by skin hypersensitivity, itching, eczema, erythema, and relapsed skin lesions (Langan et al., 2020). The prevalence of AD in adults globally, in 2018, ranged from 2.1% to 4.9% and is still increasing (Barbarot et al., 2018). Moreover, AD is the initial stage of the “atopic march,” leading to food allergy, asthma, and allergic rhinitis, which negatively affect the quality of life of patients (Spergel and Paller, 2003). In AD, levels of immunoglobulin (Ig) E, histamine, and several proinflammatory mediators, such as cytokines and chemokines, and infiltration of inflammatory cells, including mast cells and T cells, are elevated in the serum (Mansouri and Guttman-Yassky, 2015).

NC/Nga mice are the most extensively studied animal models for AD, and they exhibit symptoms similar to those of human patients with AD, for example, elevated serum levels of inflammatory factors and the infiltration of immune cells into AD-like skin lesions (Matsuda et al., 1997). These symptoms are induced by several environmental allergens. Among them, *D. farina* mites in house dust are the most common allergens that cause asthma, allergic rhinitis, and AD (Platts-Mills and Chapman, 1987; Holm et al., 1999). Repeated exposure of NC/Nga mice to the *D. farina* extract (DfE) effectively induces AD-like skin lesions in the skin when the mice are maintained under specific pathogen-free conditions (Oshio et al., 2009). Keratinocytes are the major form of epidermal cells and maintain skin homeostasis through regulation of immune cell recruitment *via* proinflammatory chemokine production (Albanesi et al., 2005). Several studies have reported that interferon-γ (IFN-γ) and tumor necrosis factor-α (TNF-α) activate inflammatory reactions in keratinocytes (Ju et al., 2009; Yang et al., 2018; Song et al., 2021). These keratinocytes produce proinflammatory chemokines, such as interleukin (IL)-8, monocyte chemoattractant protein-1 (MCP-1), thymus and activation-regulated chemokines (TARC), macrophage-derived chemokines (MDCs), and regulated on activated normal T-cell expressed and secreted (RANTES) (Werfel, 2009; Yang et al., 2013; Cha et al., 2019). Thus, controlling the production of these proinflammatory chemokines in keratinocytes can contribute to the treatment of inflammatory skin in AD.

Corticosteroids, emollients, and antihistamines are commonly used in AD. However, the long-term use of these drugs can have serious side effects (Waljee et al., 2017). Therefore, alternative medicines, including herbal extracts, have been considered in the development of novel treatment agents for AD. *S. suberectus* Dunn is a traditional Chinese herbal medicine and is used to treat anemia, rheumatism, and menoxenia (Qin et al., 2019). It has various pharmacological activities, including antitumor (Wang et al., 2011), antifungal, and antibacterial effects (Zhang et al., 2013). In addition, it has been reported to have anti-inflammatory, antioxidant, and antirheumatic effects (Li et al., 2003; Ravipati et al., 2012; Ha et al., 2013). However, to the best of our knowledge, the effects of *S. suberectus* Dunn water extract (SSWex) on AD skin lesions have not been studied. Therefore, in this study, we hypothesized that SSWex could have anti-atopic and anti-inflammatory effects on AD-like symptoms *in vivo* and *in vitro*. We investigated whether SSWex could alleviate AD-related symptoms on the skin and inflammatory reactions in the DfE-treated NC/Nga mouse model and IFN-γ/TNF-α–stimulated HaCaT cells.

## Materials and Methods

### Preparation of *S. suberectus* Dunn Water Extract

SSWex was purchased from KOC Biotech (Daejeon, South Korea). Dried *S. suberectus* Dunn (1 kg) was mixed with distilled water in a 1:10 ratio (v/w) and refluxed at 100 ± 2°C for 3 h. The extract was filtrated through a 53-μm mesh filter and finally dried in a lyophilizer to obtain a freeze-dried powder. The SSWex preparation procedure was performed as recommended by KOC Biotech. SSWex powder was stored in the herbarium of the Herbal Medicine Research Division at −20°C until further use. For *in vivo* and *in vitro* experiments, the SSWex powder was dissolved in water at a concentration of 100 mg/ml and then diluted according to each experimental condition.

### Mice and Induction of Atopic Dermatitis

**FIGURE 1:**
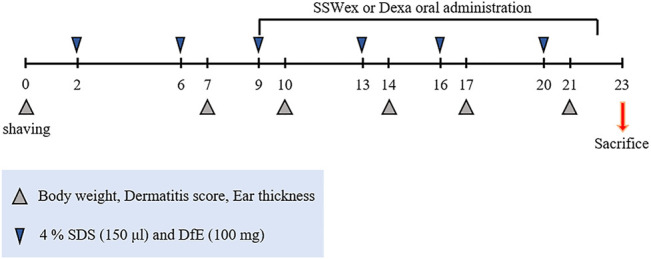
Experimental scheme for the *Dermatophagoides farinae* extract (DfE)–treated NC/Nga mouse model. The mice were sensitized with 150 μl 4% sodium dodecyl sulfate (SDS) and 100 mg DfE ointment on their shaved backs twice a week for 3 weeks. The *S. suberectus* Dunn water extract (SSWex) or dexamethasone (Dexa) was administrated orally daily from day 9 to day 22.

Eight-week-old male NC/Nga mice were purchased from Central Lab Animal Inc. (Seoul, South Korea). All mice were maintained in a specific pathogen-free environment at the Experimental Animal Center of Korea Institute of Oriental Medicine (KIOM). All animal experiments were performed in accordance with the standard of the Institutional Animal Care and Use Committee at the KIOM (approval No. 20-069). The mice were provided with *ad libitum* tap water and a standard laboratory diet (Purina 38057, Cargill Agri Purina Inc., Sungnam, South Korea). The mice were allowed to acclimatize for 1 week and further divided randomly into experimental groups (six mice/cage). The day before the start of AD induction, the fur on the back of all the mice was shaved. Thereafter, 150 μl of 4% sodium dodecyl sulfate (SDS; Sigma-Aldrich, St. Louis, MO, United States) was sprayed onto an area of over 4 cm^2^ (2 cm^2^ × 2 cm^2^) of the dorsal skin and ear surface for disruption of skin barrier. After 1 h, 100 mg of DfE (Biostir Inc., Kobe, Japan) ointment was applied onto the same areas (Pal et al., 2016; Park et al., 2020). The mice were treated with DfE twice a week for 3 weeks (Figure 1). Furthermore, 7 days after the first DfE application, SSWex (30, 100, and 300 mg/kg) or dexamethasone (Dexa; 3 mg/kg; positive control) dissolved in water was orally administered daily for 2 weeks. The NC/Nga mice were randomly divided into four groups: normal (untreated), DfE (DfE only), SSWex (DfE +3 0, 100, or 300 mg/kg SSWex), and Dexa (DfE + 3 mg/kg Dexa).

### Measurement of Ear Thickness, Dermatitis Score, and Body Weight

Ear thickness, dermatitis score, and body weight of NC/Nga mice were recorded twice a week. A digital caliper (CAS^©^, Seoul, South Korea) was used to evaluate the ear thickness. The individual scores graded as 0 (no symptoms), 1 (mild), 2 (moderate), or 3 (severe) were calculated for each of the four symptoms (scarring/dryness, erythema/hemorrhage, edema, and excoriation/erosion). All dermatitis severity scores were quantified as the sum of all individual scores. The body weights of the mice were measured for monitoring.

### Analysis of Serum Immunoglobulin E, Histamine, and Proinflammatory Chemokines

The serum samples were collected from NC/Nga mice that were killed on the last day of the experiment and then stored at −80°C until analysis. The LBIS Mouse Immunoglobulin E (IgE) ELISA Kit (Fujifilm, Shibukawa, Japan) was used to determine the total levels of IgE. Histamine levels were measured using Histamine Research ELISA™ (LDN, Nordhorn, Germany). The serum levels of total TARC, MDC, and RANTES were detected using mouse ELISA kits from R&D Systems (Minneapolis, MN, United States). These experiments were conducted according to the manufacturer’s instructions.

### Histopathological Analysis and Immunohistochemistry

The ear and back skin tissues of the mice were removed, fixed with 10% formaldehyde, embedded in paraffin, and serially sectioned into 4–6-μm sections using a microtome (Leica Biosystems, Newcastle, United Kingdom). The tissues were mounted on slides and stained with hematoxylin/eosin solution (Sigma-Aldrich) or toluidine blue (Toluidine Blue Stain Kit, VitroVivo Biotech, Rockville, MA, United States). For immunohistochemical staining, the slide-mounted tissues were incubated overnight at 4°C with CD3 primary antibodies (1:150 dilution; cat.no. ab16669, Abcam, Cambridge, United Kingdom) and processed using a XT System Benchmark autostainer (Ventana Medical System, Tucson, AZ, United States) according to the manufacturer’s instruction. The cells positive for CD3 and toluidine blue staining as measured from the tissue sections were analyzed using Solution for Automatic Bio-Image Analysis software (Ebiogen, Seoul, South Korea).

### Cells and Reagents

Human keratinocyte HaCaT cells were purchased from Elabscience (Catalog No. EP-CL-0090, Houston, TX, United States). The cells were cultured in a high-glucose-containing Dulbecco’s modified Eagle’s medium (Hyclone, Logan, UT, United States) supplemented with 1% penicillin/streptomycin (Gibco-BRL, Gaithersburg, MD, United States) and heat-inactivated 10% fetal bovine serum (Gibco-BRL) in a humidified incubator containing 5% CO_2_ at 37°C. Recombinant human IFN-γ, TNF-α, Alexa 594 goat anti-rabbit antibody (cat.no. A-11037), Alexa 488 goat anti-rabbit antibody (cat.no. A-11034), and Alexa 488 goat anti-mouse antibody (cat.no. A-10680) were purchased from Thermo Fisher Scientific (Waltham, MA, United States). DRAQ5™, p-IκBα (cat.no. 2859), IκBα (cat.no. 9242), p65 (cat.no. 8242), β-actin (cat.no. 3700), p-JNK (cat.no. 9251), JNK (cat.no. 9252), p-ERK (cat.no. 9101), ERK (cat.no. 9102), p-p38 (cat.no. 9211), p38 (cat.no. 9212), p-STAT1 (Tyr) (cat.no. 9167), p-STAT1 (Ser) (cat.no. 9177), and STAT1 (cat.no. 9172) antibodies were purchased from Cell Signaling Technology (Beverly, MA, United States). PCNA (cat.no. sc-56), p50 (cat.no. sc-8414), secondary horseradish peroxidase (HRP)–conjugated anti-mouse (cat.no. sc-2357), and anti-rabbit (cat.no. sc-516102) antibodies were purchased from Santa Cruz Biotechnology (Santa Cruz, CA, United States).

### Cell Cytotoxicity

Cell cytotoxicity was determined using the CellTiter 96^®^ AQueous One Solution Cell Proliferation Assay kit (Promega, Madison, WI, United States). HaCaT cells were allowed to attach to 96-well plates overnight, treated with various concentrations of SSWex (0–500 μg/ml), and incubated for 24 h. Furthermore, a prewarmed 20 μl assay reagent was added to each well, and the cells were incubated at 37°C for 1 h. The absorbance was detected at 490 nm using a Synergy HTX Multi-Mode Reader (BioTek, Winooski, VT, United States).

### Analysis of Secreted Proinflammatory Chemokines

The cells in the 96-well plate were preincubated with SSWex at indicated concentrations (50, 100, and 300 μg/ml) for 1 h and stimulated with 10 ng/ml of IFN-γ/TNF-α for 24 h at 37°C. After stimulation, the supernatant was collected and centrifuged at 4,000 rpm for 5 min to remove the particulate matter. The levels of secreted proinflammatory chemokines, such as MDC and RANTES, in stimulated HaCaT cells were detected using human ELISA kits (R&D Systems), in accordance with the manufacturer’s instructions. The released TARC, MCP-1, and IL-8 were calculated using the LEGENDplex™ Human Proinflammatory Chemokine Panel (BioLegend, San Diego, CA, United States). The bead-based immunoassay was performed according to the manufacturer’s instructions. The stained-bead samples were detected using a BD LSRFortessa™ Flow Cytometer (BD Biosciences, San Jose, CA, United States) and analyzed using BD CellQuest™ software. Data were formalized using LEGENDplex™ Software v8.0 (VigeneTech Inc., Carlisle, MA, United States).

### Western Blot Analysis

HaCaT cells were pretreated with 300 μg/ml SSWex for 1 h and incubated with 10 ng/ml IFN-γ/TNF-α at 37°C for the indicated times (0–30 min). The cell lysates were prepared using ice-cold radioimmunoprecipitation assay buffer (Biosesang, Seongnam, South Korea) containing Halt™ Protease and Phosphatase Inhibitor Cocktail (Thermo Fisher Scientific). The lysates were centrifuged at 13,000 rpm for 10 min at 4°C. Quantification of the collected proteins in supernatants was performed using the Pierce™ BCA assay kit (Thermo Fisher Scientific). Total proteins (20 μg) were separated using 4%–15% Mini-PROTEAN TGX Precast Protein Gels (Bio-Rad, Hercules, CA, United States) by electrophoresis and further transferred to Fluoro Trans^®^ PVDF Membrane (Pall Corporation, Dreieich, Germany). The membranes were blocked with 5% skim milk (Sigma-Aldrich) or 3% bovine serum albumin (BSA; MP Biomedicals, Irvine, CA, United States) in tris-buffered saline with 1% tween-20 (TBST) for 2 h at 4°C, followed by overnight incubation with primary antibodies (1:1,000 dilution) in blocking solutions. After incubation with secondary antibodies (horseradish peroxidase–conjugated anti-IgG) diluted in blocking buffer at a ratio of 1:2500 for 1 h, the signals on the membranes were developed using the Super Signal West Femto Chemiluminescent Substrate (Thermo Fisher Scientific). Protein detection was performed using the ChemiDoc Imaging System (Bio-Rad).

### Nuclear Fraction

HaCaT cells were pretreated with various concentrations of SSWex for 1 h, followed by stimulation with 10 ng/ml IFN-γ/TNF-α for 1 h at 37°C. The stimulated cells were immediately washed twice with ice-cold PBS and harvested. Nuclear proteins were isolated using NE-PER^®^ Nuclear and Cytoplasmic Extraction Reagents (Pierce Biotechnology, Rockford, IL, United States) according to the manufacturer’s protocol.

### Immunofluorescence Staining

HaCaT cells (6 × 10^5^/dish) were seeded in a 12-mm Nunc Glass Base dish (Thermo Fisher Scientific). The cells were preincubated with 300 μg/ml of SSWex for 1 h, followed by stimulation with 10 ng/ml IFN-γ/TNF-α for 1 h at 37°C. The stimulated cells were washed twice with PBS and fixed in 3% paraformaldehyde (diluted with PBS) for 20 min at 4°C. The fixed cells were washed four times with 0.1% TritonX-100 buffer for 10 min, blocked with 3% BSA (diluted with 0.1% TritonX-100 buffer) for 1 h at room temperature, and incubated with the primary antibodies (1:500) overnight at 4°C. The primary antibodies used here were the same as those used in Western blot analysis. Subsequently, the cells were incubated with Alexa Fluor 594 or 488 anti-rabbit or mouse IgG secondary antibody (1:500) for 2 h at 4°C. The nuclei were stained using DRAQ5™ in blocking buffer. After incubation of 15 min, the cells were acquired using an FV10i confocal microscope (Olympus, Tokyo, Japan).

### High-Performance Liquid Chromatography

To perform simultaneous determination and quantitative analysis of the four reference compounds [gallic acid, (+)-catechin, procyanidin B2, and epicatechin] in SSWex, a Waters e2695 liquid chromatography system (Waters Co., Milford, MA, United States) equipped with a Waters 2998 photodiode array detector was used. Empower software (version 3, Waters Co.) was used for data acquisition and processing. The four compounds were separated on a Phenomenex Luna C18 column (250 mm × 4.6 mm; particle size 5 μm; Phenomenex, Torrance, CA, United States) and detected at 280 nm. The mobile phase was 0.1% aqueous acetic acid (A) and acetonitrile (B) in a gradient elution mode. The gradient elution was set as follows: 0–10 min, 5%–15% B; 10–30 min, 15%–20% B; and 30–40 min, 20%–30% B. After each analysis, an 8-min wash with acetonitrile was performed, and a further equilibration time of 8 min was observed to return to the initial mobile phase composition. The flow rate of the mobile phase was 1.0 ml/min, and the injection volume was 20 μl. SSWex was dissolved in methanol (10 mg/ml) and filtered through a 0.2-μm syringe filter to prepare the sample solutions for quantitative analysis of the four compounds. The four reference compounds with purity > 98% were used for analysis. Gallic acid was purchased from ChemFaces Biochemical (Wuhan, China), and all other compounds [(+)-catechin, procyanidin B2, and epicatechin] were obtained from Biopurify Phytochemicals (Chengdu, China).

### Statistical Analyses

All results from more than three independent experiments are indicated as the means ± standard error of mean (S.E.M.). Statistical analyses were performed using GraphPad Prism version 8.0 (GraphPad Software, San Diego, CA, United States) by ordinary one-way analysis of variance. Between-group comparisons were performed using Tukey’s *post hoc* test to calculate statistical significance (*p* < 0.05).

## Results and Discussion

### *S. suberectus* Dunn Water Extract Inhibits the Clinical Severity of Atopic Dermatitis–Like Skin Symptoms and Histological Features in *D. farina* Extract–Treated NC/Nga Mice

**FIGURE 2:**
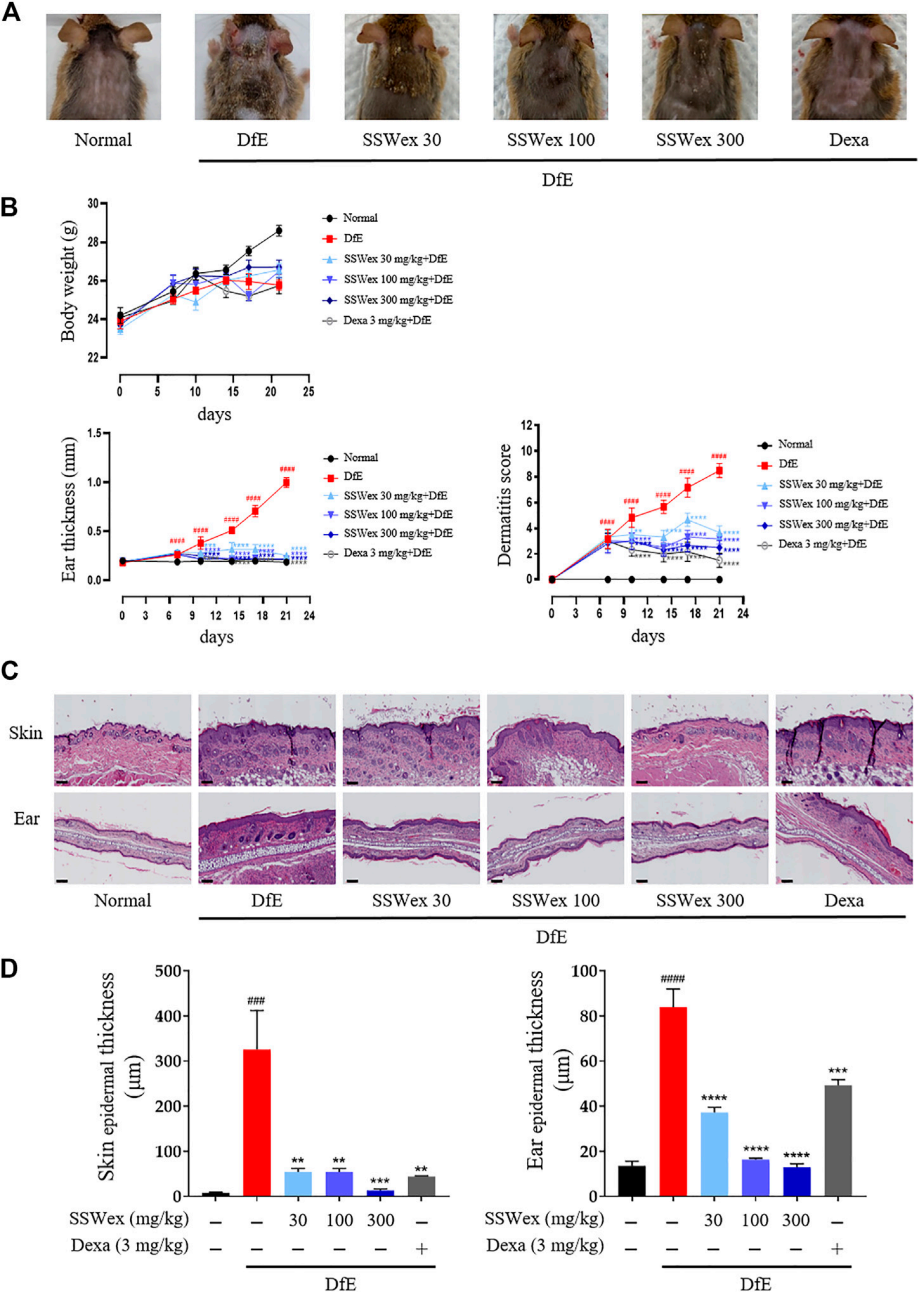
Effect of SSWex on DfE-induced clinical and histopathological features of the AD-like skin lesions in NC/Nga mice. **(A)** Photographic images of the backs of mice from each group on the last day of the experiment. **(B)** Body weight, ear thickness, and dermatitis scores were measured twice a week for 3 weeks. **(C)** Skin and ear tissue sections were stained with hematoxylin and eosin (H&E). The stained sections were observed under a microscope at ×100 magnification. Scale bar = 100 μm. **(D)** Skin and ear epidermis thicknesses of the DfE-treated NC/Nga mice were analyzed and represented as a graph. Values are presented as the mean ± S.E.M (*n* = 6). ^#^ *p* < 0.05, ^##^ *p* < 0.005, ^###^ *p* < 0.0005, and ^####^ *p* < 0.0001 vs. the normal group; *p < 0.05, **p < 0.005, ***p < 0.0005, and ****p < 0.0001 vs. the DfE group.

To investigate the effects of SSWex on AD-like symptoms in NC/Nga mice, DfE or Dexa was administrated twice a week for 3 weeks. Pathological symptoms of AD, such as skin swelling, erythema, cornification, exudation, dry skin, and increased ear thickness, were observed in DfE-treated NC/Nga mice (Yang et al., 2013; Kang et al., 2017). The application of SSWex significantly relieved AD-like symptoms, including ear thickness and dermatitis score, in DfE-treated NC/Nga mice. No difference in body weight was observed among the DfE-treated groups (Figures 2A,B). Dexa 3 mg/kg was used as a positive control (Kim et al., 2009). Repeated application of DfE to NC/Nga mice causes various symptoms of AD, such as epidermal thickening of the skin (Kang et al., 2017). The increase in epidermal thickness was due to epidermal proliferation, which is pathologically activated by the differentiation of keratinocytes in inflammatory skin lesions (Limandjaja et al., 2017). To histologically evaluate the effects of SSWex on AD-like skin lesions, we performed skin histological analysis using hematoxylin and eosin staining. Epidermis thickness increased in the DfE group compared with that in the normal group; however, SSWex treatment significantly reduced the epidermis thickness in the dorsal skin and ear tissue (Figures 2C,D). These results indicated that SSWex may alleviate AD-like symptoms and histological features of skin and ear lesions in DfE-treated NC/Nga mice.

### *S. suberectus* Dunn Water Extract Reduces the Infiltration of Mast Cells and T Cells in Skin Lesions in *D. farina* Extract–Treated NC/Nga Mice

**FIGURE 3:**
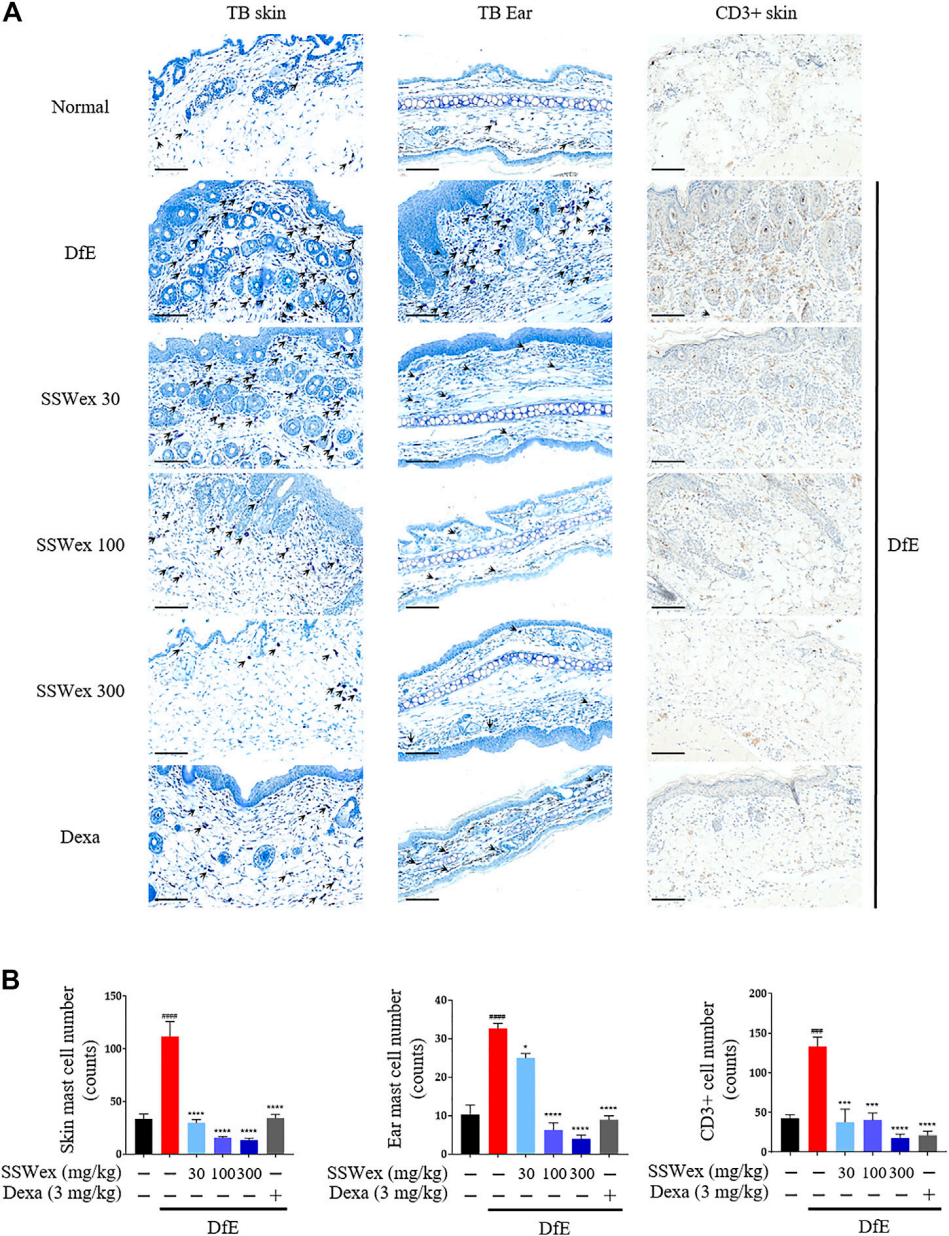
Effect of SSWex on the infiltration of mast and T cells in skin lesions of DfE-treated NC/Nga mice. **(A,B)** Sectioned dorsal skin and ears were stained with toluidine blue to evaluate mast cell infiltration. Dorsal skin tissue sections were immunostained with anti–CD3 antibodies to confirm T-cell infiltration. The stained sections were observed under a microscope at ×400 magnification. Scale bar = 100 μm. Data are represented as the mean ± S.E.M. (*n* = 6). ^#^ *p* < 0.05, ^##^ *p* < 0.005, ^###^ *p* < 0.0005, and ^####^ *p* < 0.0001 vs. the normal group; *p < 0.05, **p < 0.005, ***p < 0.0005, and ****p < 0.0001 vs. the DfE group.

Patients with AD have increased infiltration of immune cells, such as mast and T cells, in AD skin lesions (Leung et al., 2004; Peng and Novak, 2015). Mast cells are innate immune cells that are thought to be involved in allergic diseases, including AD; they recognize specific antigens through high-affinity receptors for IgE (FcεRI) (Hofmann and Abraham, 2009). Mast cells are believed to be involved in the pathogenesis of AD through a wide range of proinflammatory mediators secreted from FcεRI-activated mast cells, along with an elevation in IgE levels and the number of mast cells (Kawakami et al., 2009). In addition, the sensory nerve density of the epidermis and dermis increases in AD-like skin lesions (Gupta and Harvima, 2018). Therefore, we examined the number of mast cells in the skin and ear of DfE-treated NC/Nga mice. Toluidine blue staining revealed that the number of mast cells increased in the DfE group compared with that in the normal group, whereas SSWex administration reduced the number of mast cells in AD-like lesions in the dorsal skin and ear (Figure 3). In addition, to investigate the effect of SSWex on T-cell infiltration in AD-like skin lesions, we analyzed the number of CD3^+^ (T-cell marker) cells using immunohistochemistry. We observed an increased number of CD3^+^ cells in AD-like skin lesions, consistent with previous studies (Park et al., 2012; Yang et al., 2014a). SSWex treatment decreased T-cell infiltration in DfE-induced AD on the skin (Figure 3). AD is caused by an imbalance between T-helper (Th) 1 and Th2 cells (Bieber, 2010). Among the infiltrating T cells, Th2 cells are one of the major cell types involved in AD development (Purushothaman et al., 2018). Although Th2-mediated responses are more prominent in the acute AD phase, Th1-mediated responses are more prominent in the chronic AD phase (Leung et al., 2004). Therefore, reducing the infiltration of mast and T cells in skin lesions in AD is important. Our results demonstrated that SSWex treatment could reduce this infiltration and thus alleviate skin lesions in DfE-treated NC/Nga mice.

### *S. suberectus* Dunn Water Extract Inhibits *D. farina* Extract–Induced Serum Immunoglobulin E, Histamine, and Proinflammatory Chemokine Production in NC/Nga Mice

**FIGURE 4:**
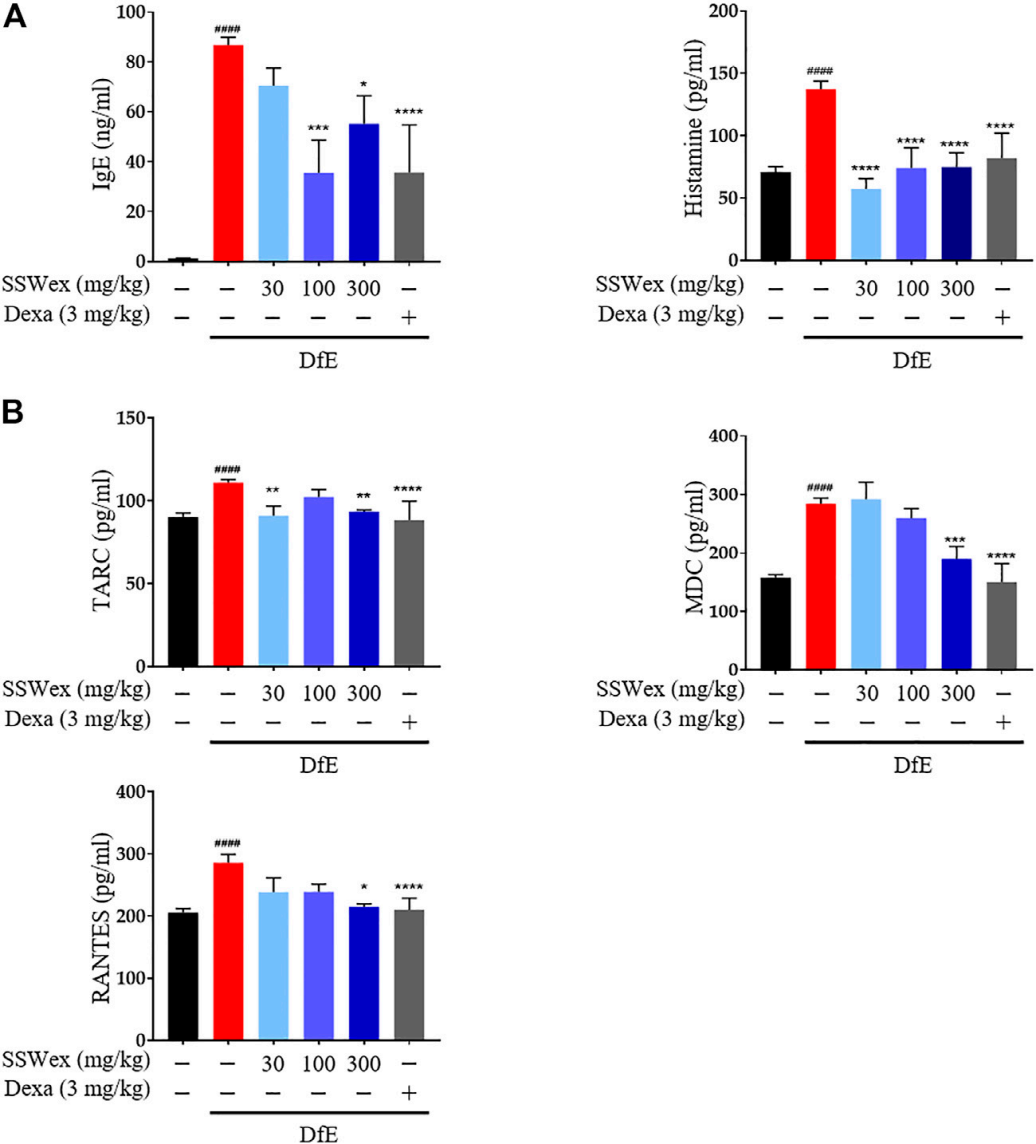
Effect of SSWex on serum immunoglobulin E (IgE), histamine, and proinflammatory chemokine levels in DfE-induced atopic dermatitis (AD)–like skin lesions in NC/Nga mice. The serum samples were collected on the last day of the experiment. The serum samples were diluted ×100 times and analyzed. **(A)** Total IgE and histamine levels in serum were detected using ELISA. **(B)** Levels of proinflammatory chemokines (MDC, RANTES, and TARC) were determined using ELISA. Data are expressed as the mean ± S.E.M. (*n* = 6). ^#^ *p* < 0.05, ^##^ *p* < 0.005, ^###^ *p* < 0.0005, and ^####^ *p* < 0.0001 vs. the normal group; *p < 0.05, **p < 0.005, ***p < 0.0005, and ****p < 0.0001 vs. the DfE group.

IgE is an important component of allergic diseases and is closely associated with Th2 immune response (Leung, 1993). It leads to the secretion of various allergic mediators, including histamines and cytokines, by binding to mast cells (O’Mahony et al., 2011). Particularly, the IgE–mast cell–histamine axis has been well-understood for decades, and this pathway is known to cause AD-associated itching disorders (Yang and Kim, 2019). The inhibitory effects of SSWex on AD-induced serum IgE and histamine release were measured using ELISA. DfE-induced AD-like skin inflammation was accompanied by an increase in serum IgE and histamine levels; however, SSWex treatment inhibited this increase in DfE-treated NC/Nga mice (Figure 4A).

Proinflammatory chemokines play a major role in various processes of AD progression, such as immune cell activation, differentiation, and infiltration of inflammatory sites (Sebastiani et al., 2002). Proinflammatory chemokines can be produced by various immune cells, including mast cells, T cells, dendritic cells, keratinocytes, and eosinophils, activated by various stimuli in AD (Nedoszytko et al., 2014; Pucheu-Haston et al., 2015). Among these chemokines, TARC and MDC are CC chemokine receptor type (CCR) 4 ligands, which play important roles in the infiltration of Th2 cells into AD skin lesions (Oshio et al., 2009). In addition, RANTES is one of the CCR ligands that play an active role in regulating the infiltration and activation of immune cells, including Th2 and mast cells, in AD-related inflammatory tissues (Alam, 1997). TARC, MDC, and RANTES are highly expressed in AD patients and mouse models (Hashimoto et al., 2006; Oshio et al., 2009; Lim et al., 2014; Ko et al., 2019). Thus, the role of SSWex in inhibiting the production of these proinflammatory chemokines in DfE-treated NC/Nga mice was evaluated. SSWex treatment significantly reduced the serum levels of TARC, MDC, and RANTES (Figure 4B). These results support the inhibitory action of SSWex on the infiltration of immune cells, including mast and T cells. In this study, *in vivo* data demonstrated that SSWex administration improved AD-like skin lesions in NC/Nga mice *via* alleviating multiple DfE-induced events.

### *S. suberectus* Dunn Water Extract Suppresses the Production of Proinflammatory Chemokines in IFN-γ/TNF-α–Stimulated HaCaT Cells

Epidermal keratinocytes in AD skin lesions play a major role in the immune response through the secretion of inflammatory mediators, such as proinflammatory chemokines. The inflammatory responses of synergistically activated keratinocytes by IFN-γ/TNF-α are mainly used in studies for inflammatory skin diseases, including AD (Yang et al., 2015; Lee et al., 2018; Lee et al., 2020). The keratinocytes activated by IFN-γ/TNF-α release proinflammatory chemokines, such as TARC, MDC, and RANTES, which play an important role in the infiltration of Th2 cells into AD skin lesions (Yang et al., 2015). In addition, co-stimulation of IFN-γ/TNF-α is known to release monocyte- and neutrophil-specific chemokines, such as MCP-1 and IL-8, in inflamed keratinocytes. The release of these chemokines is considered to play a major role in the recruitment and accumulation of inflammatory cells in skin inflammatory diseases (Sebastiani et al., 2002; Cha et al., 2019). Therefore, inhibition of the release of these proinflammatory chemokines in keratinocytes of AD skin lesions is considered important in AD treatment.

**FIGURE 5:**
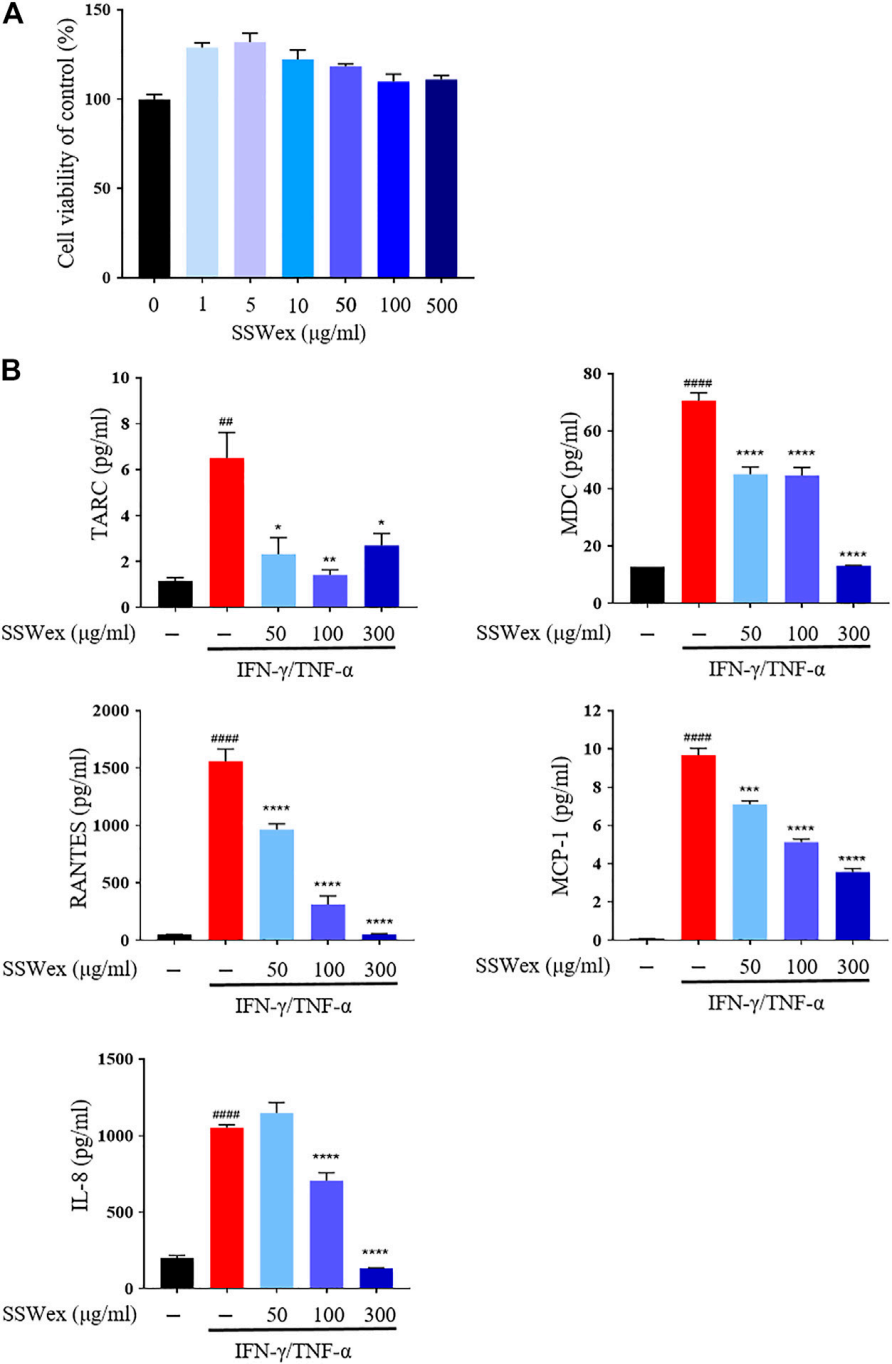
Effects of SSWex on proinflammatory cytokines and chemokines in IFN-γ-/TNF-α–induced HaCaT cells. **(A)** HaCaT cells treated with SSWex at different concentrations (0–500 μg/ml) for 24 h. Cell viability was determined using a cell proliferation assay kit. **(B)** HaCaT cells were pretreated with SSWex for 1 h and then stimulated with 10 ng/ml of IFN-γ/TNF-α for 24 h. Secreted MDC and RANTES were measured *via* ELISA. Secreted TARC, MCP-1, and IL-8 were detected using the bead-based immunoassay. The data are shown as the mean ± S.E.M. of three independent experiments. ^#^ *p* < 0.05, ^##^ *p* < 0.005, ^###^ *p* < 0.0005, and ^####^ *p* < 0.0001 vs. untreated; *p < 0.05, **p < 0.005, ***p < 0.0005, and ****p < 0.0001 vs. IFN-γ/TNF-α.

To determine the cytotoxicity of SSWex, HaCaT cells were incubated with SSWex in a dose-dependent manner for 24 h. SSWex appeared to be non-toxic till a level of 500 μg/ml (Figure 5A). To investigate the inhibitory effects of SSWex on IFN-γ/TNF-α–induced proinflammatory chemokine production, HaCaT cells were pretreated with SSWex for 1 h and further stimulated with IFN-γ/TNF-α for 24 h. The proinflammatory chemokines released in the cell culture medium supernatant were analyzed using ELISA or bead-based immunoassay. SSWex (50–300 μg/ml) significantly inhibited the production of IFN-γ/TNF-α–induced TARC, MDC, RANTES, MCP-1, and IL-8 in HaCaT cells (Figure 5B). Therefore, the *in vitro* anti-atopic effects of SSWex were confirmed.

### *S. suberectus* Dunn Water Extract Inhibits IFN-γ/TNF-α–Induced MAPK/STAT1/NF-κB Activation in HaCaT Cells

IFN-γ and TNF-α activate the extracellular signal–regulated kinases (ERK), c-Jun N-terminal kinases (JNK), and p38 mitogen–activated protein kinase (MAPK) pathways in various cell types, including keratinocytes (Sung et al., 2012; Jeong et al., 2016). The MAPKs regulate the synthesis of proinflammatory molecules, such as chemokines, in AD through their intracellular signaling pathways, including signal transducer and activator of transcription 1 (STAT1) and nuclear factor kappa B (NF-κB) (Kwon et al., 2012; Park et al., 2015a; An et al., 2017). STAT1 and NF-κB are important transcription factors in the immune system and are stimulated by IFN-γ/TNF-α to produce proinflammatory chemokines in HaCaT cells (Park et al., 2015b; Yang et al., 2018). Upon stimulation with IFN-γ/TNF-α, STAT1 is activated, phosphorylated, and further translocated to the nucleus where it can activate the target genes by binding to promoters. Similarly, activation of NF-κB by the phosphorylation and degradation of inhibitor kappa B-alpha (IκB-α) causes translocation of NF-κB (p65 and p50) to the nucleus where it can activate the target genes by binding to promoters (Sung et al., 2012). Thus, inhibition of the MAPK/STAT1/NF-κB signaling pathway can alleviate the symptoms of AD and is considered an important strategy in the development of novel therapeutics for AD.

**FIGURE 6:**
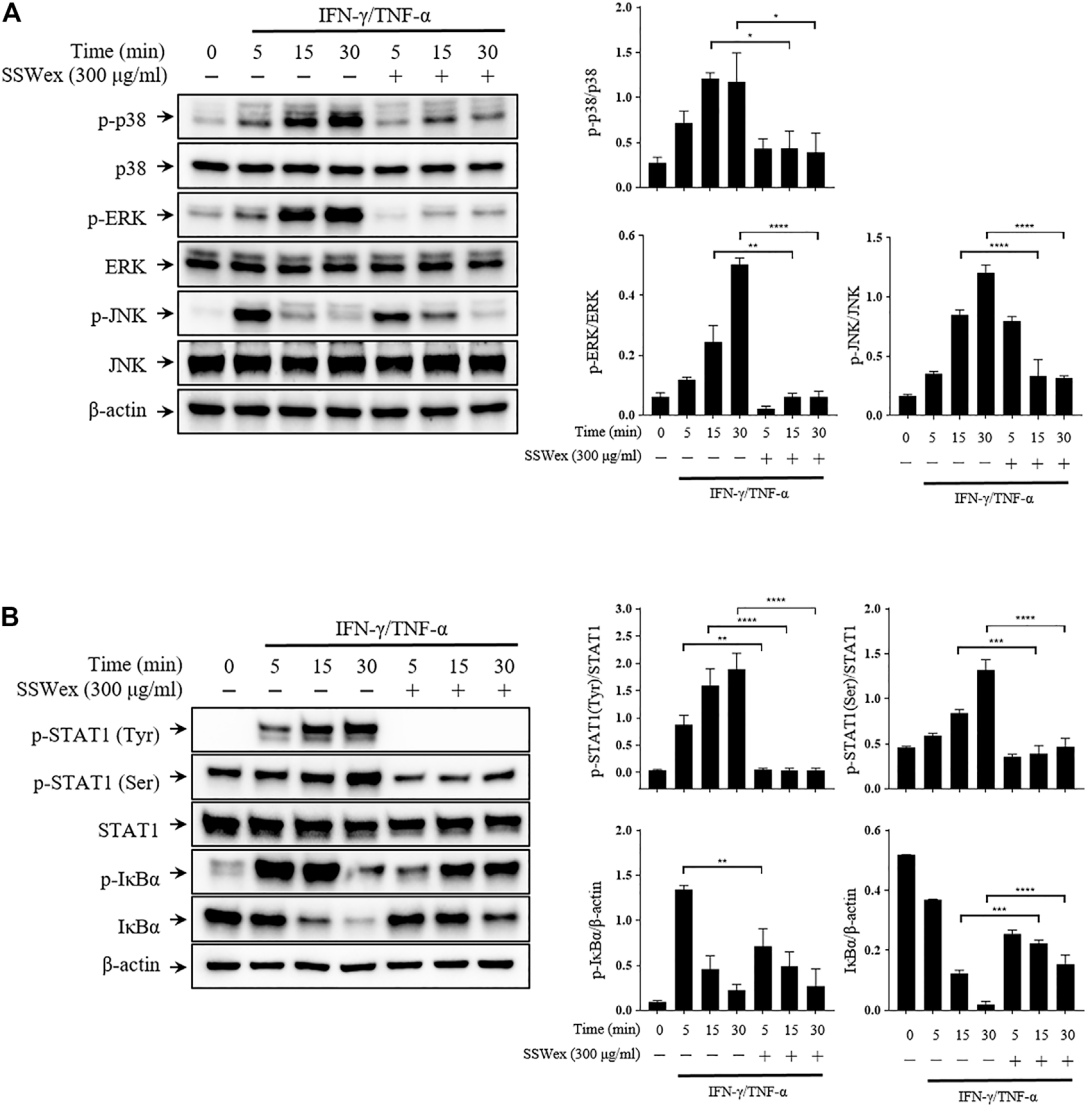
Effect of SSWex on MAPK/NF-κB/STAT1 activation by IFN-γ-/TNF-α in HaCaT cells. The cells were preincubated with SSWex for 1 h and further stimulated with IFN-γ/TNF-α for various time intervals (0, 5, 15, and 30 min). Using a Western blot analysis, **(A)** the phosphorylated and total MAPK (p38, ERK, and JNK) protein levels were quantified and **(B)** phosphorylation or degradation of IκBα and STAT1 proteins was detected in the cells. Quantified Western blot data are shown as the means ± S.E.M. of three separate experiments. *p < 0.05, **p < 0.005, ***p < 0.0005, and ****p < 0.0001 vs. IFN-γ/TNF-α.

To investigate the molecular mechanism of the inhibitory effect of SSWex in IFN-γ-/TNF-α–stimulated HaCaT cells, we first determined whether SSWex inhibits the activation of the MAPK signaling pathway using Western blot analysis. SSWex 300 μg/ml inhibited the IFN-γ-/TNF-α–induced phosphorylation of p38, ERK, and JNK in HaCaT cells (Figure 6A). In addition, SSWex treatment inhibited the IFN-γ-/TNF-α–induced phosphorylation of STAT1 and IκBα and degradation of IκBα in HaCaT cells (Figure 6B).

**FIGURE 7:**
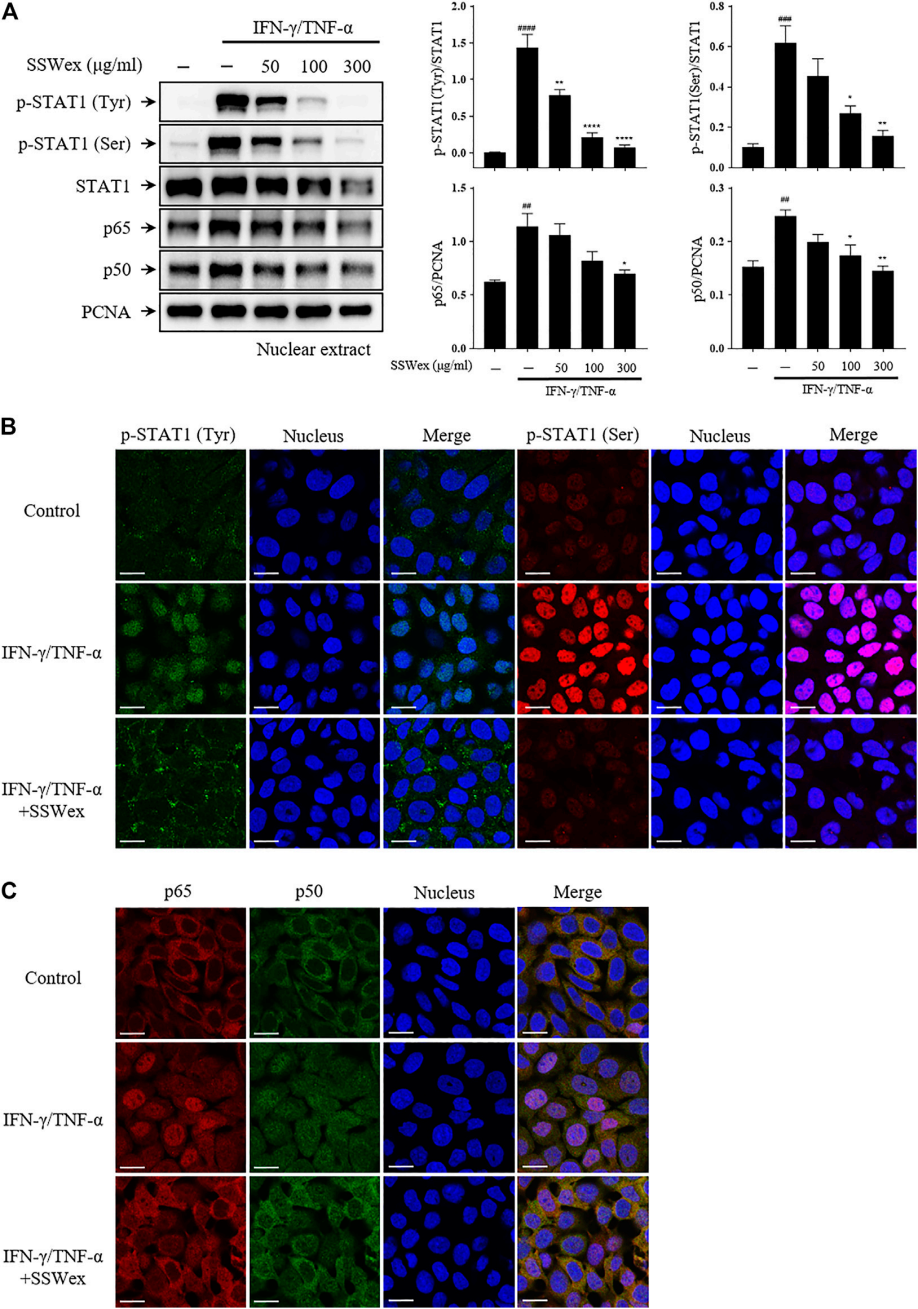
Effect of SSWex on IFN-γ-/TNF-α–induced NF-κB and STAT1 translocation to the nucleus in HaCaT cells. The cells were pretreated with 50, 100, or 300 μg/ml SSWex for 1 h and further incubated with 10 ng/ml IFN-γ/TNF-α for 1 h. **(A)** For Western blot analysis, nuclear fractionation was performed to confirm p-STAT1, STAT1, and NF-κB (p65 and p50) subunits in the nucleus. β-Actin and PCNA were used as loading controls. **(B,C)** Quantified Western data are shown as the mean ± S.E.M. of three independent experiments. ^#^ *p* < 0.05, ^##^ *p* < 0.005, ^###^ *p* < 0.0005, and ^####^ *p* < 0.0001 vs. untreated; *p < 0.05, **p < 0.005, ***p < 0.0005, and ****p < 0.0001 vs. IFN-γ/TNF-α. The nuclear translocation of p-STAT1 (Tyr; green and Ser; red) and NF-κB (p65; red and p50; green) was detected by immunofluorescence combined with DRAQ5™ (blue) staining for the nucleus (scale bar = 20 μm).

Furthermore, we studied the effect of SSWex on the nuclear translocation of STAT1 and NF-κB using Western blot after nuclear fractionation. SSWex treatment suppressed the IFN-γ-/TNF-α–induced nuclear translocation of p-STAT1, p65, and p50 in a dose-dependent manner (Figure 7A). This inhibitory effect of SSWex was also demonstrated by immunofluorescence staining (Figures 7B,C). These results demonstrated that SSWex inhibited the activation of keratinocytes by inhibiting proinflammatory chemokine production *via* regulation of the MAPK/STAT1/NF-κB signaling pathway.

### High-Performance Liquid Chromatography Analysis of Four Compounds in the *S. suberectus* Dunn Water Extract

**FIGURE 8:**
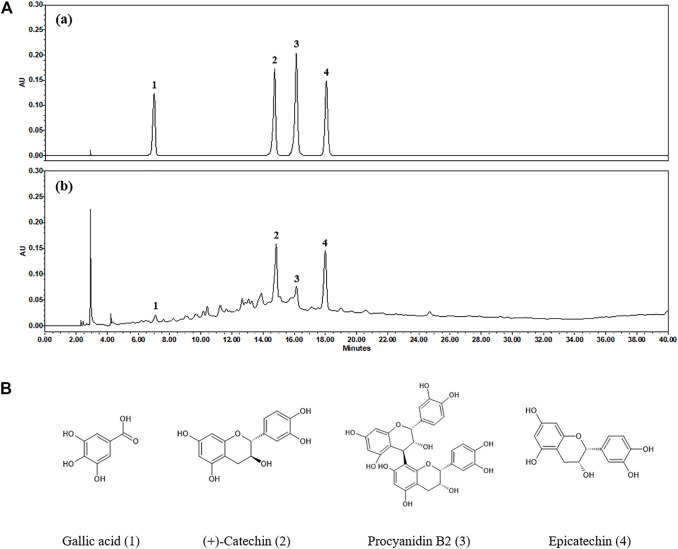
**(A)** HPLC chromatograms of the standard mixture **(a)** and SSWex **(b)** at 280 nm. **(B)** Chemical structures of the four compounds in SSWex: gallic acid (1), (+)-catechin (2), procyanidin B2 (3), and epicatechin (4).

High-performance liquid chromatography (HPLC) analysis was performed with an analytical method established to separate the marker compounds in SSWex. The four compounds in SSWex were detected simultaneously: gallic acid, (+)-catechin, procyanidin B2, and epicatechin with the retention times of 7.11, 14.85, 16.15, and 17.99 min, respectively. The representative HPLC chromatogram is shown in Figure 8A, and the chemical structure of each compound is shown in Figure 8B. The coefficients of determination (r^2^) calculated from the calibration curves of the four compounds were all ≥0.9997, indicating good linearity. The quantitative analysis revealed that the amounts of gallic acid, (+)-catechin, procyanidin B2, and epicatechin in SSWex were 0.409, 6.129, 1.884, and 3.951 mg/g, respectively (Table 1).

**TABLE 1:** Calibration curves and contents of the four compounds in *Spatholobus suberectus* Dunn.

Compound	Linear range (μg/ml)	Regression equation	*r* ^2^	Content (mg/g)	
				Mean	SD
Gallic acid	2.50–160.00	*y* = 40290*x* − 68434	0.9997	0.409	0.001
(+)-Catechin	3.44–220.00	*y* = 14186*x* − 20060	0.9998	6.129	0.112
Procyanidin B2	3.44–220.00	*y* = 14714*x* − 6332.8	0.9999	1.884	0.009
Epicatechin	3.91–250.00	*y* = 29502*x* − 3311.9	0.9999	3.951	0.005

Gallic acid, one of the most important polyphenols, has been found in many plants and fruits, such as grapes, strawberries, bananas, gallnuts, and green tea (Zuo et al., 2002; Yeganeh Ghotbi and bin Hussein, 2010; Bhat et al., 2016). It possesses antitumorigenic and anti-inflammatory activities (Faried et al., 2007; Kaur et al., 2009). Moreover, it can exhibit anti-allergic inflammatory activity on crucial effector cells in allergic inflammation, such as basophils, eosinophils, and dendritic cells (Liu et al., 2013; Chan et al., 2015; Tsang et al., 2016). Catechins, including (+)-catechin and epicatechin, are widely present in foods and herbs, such as berries, grapes, cacaos, and apples. They are particularly present in high amounts in tea (Isemura, 2019). Catechins have many beneficial properties for human health, such as antimicrobial, anticancer, ROS regulatory, anti-aging, antioxidant, anticardiovascular disease, and anti-inflammatory activities (Isemura, 2019; Nakano et al., 2019). Procyanidin B2 is a phenolic compound and is mainly found in grapes, apples, blueberries, cocoa, and tea (Su et al., 2018; Xiao et al., 2018). Procyanidin B2 is reported to exhibit various pharmacological activities, including antioxidant, antitumor, and anti-inflammatory properties (Sakano et al., 2005; Suda et al., 2013; Yang et al., 2014b). Among these activities of procyanidin B2, anti-inflammatory effects occur through regulation of various inflammatory mediators, including cytokines, chemokines, and nitric oxide. Procyanidin B2 regulates these inflammatory mediators *via* the regulation of MAPK/NF-κB activity (Martinez-Micaelo et al., 2012a; Martinez-Micaelo et al., 2012b). Therefore, the anti-inflammatory effects of these four compounds could be responsible for the anti-inflammatory action of SSWex. In addition, the anti-atopic effects of gallic acid (Hu and Zhou, 2021) and epicatechin (Song et al., 2021) have already been reported. Since there are no reports of the anti-atopic effects of (+)-catechin or procyanidin B2, we will evaluate these in subsequent studies.

## Conclusion

The topical application of SSWex suppressed AD symptoms in skin lesions in DfE-treated NC/Nga mice. SSWex inhibited immune cell infiltration in AD-like skin lesions and increase in AD-related serum parameters, such as IgE, histamine, and proinflammatory chemokines. Moreover, we observed that SSWex regulated the expression of proinflammatory chemokines *via* the MAPK/STAT1/NF-κB pathway in IFN-γ-/TNF-α–stimulated HaCaT cells. Our study provides evidence of the potential of SSWex as a novel agent or food supplement for the prevention and treatment of AD.

## Data Availability

The raw data supporting the conclusion of this article will be made available by the authors, without undue reservation.
